# Dysregulation of Mitochondrial Dynamics and the Muscle Transcriptome in ICU Patients Suffering from Sepsis Induced Multiple Organ Failure

**DOI:** 10.1371/journal.pone.0003686

**Published:** 2008-11-10

**Authors:** Katarina Fredriksson, Inga Tjäder, Pernille Keller, Natasa Petrovic, Bo Ahlman, Camilla Schéele, Jan Wernerman, James A. Timmons, Olav Rooyackers

**Affiliations:** 1 Department of Anesthesiology and Intensive Care, Karolinska Institute, Karolinska University Hospital, Stockholm, Sweden; 2 Translational Biomedicine, Heriot-Watt University, Edinburgh, Scotland, United Kingdom; 3 Department of Surgery, CLINTEC, Karolinska Institute, Ersta hospital, Stockholm, Sweden; 4 The Wenner-Gren Institute, The Arrhenius Laboratories, Stockholm University, Stockholm, Sweden; Instituto Oswaldo Cruz and FIOCRUZ, Brazil

## Abstract

**Background:**

Septic patients treated in the intensive care unit (ICU) often develop multiple organ failure including persistent skeletal muscle dysfunction which results in the patient's protracted recovery process. We have demonstrated that muscle mitochondrial enzyme activities are impaired in septic ICU patients impairing cellular energy balance, which will interfere with muscle function and metabolism. Here we use detailed phenotyping and genomics to elucidate mechanisms leading to these impairments and the molecular consequences.

**Methodology/Principal Findings:**

Utilising biopsy material from seventeen patients and ten age-matched controls we demonstrate that neither mitochondrial *in vivo* protein synthesis nor expression of mitochondrial genes are compromised. Indeed, there was partial activation of the mitochondrial biogenesis pathway involving NRF2α/GABP and its target genes TFAM, TFB1M and TFB2M yet clearly this failed to maintain mitochondrial function. We therefore utilised transcript profiling and pathway analysis of ICU patient skeletal muscle to generate insight into the molecular defects driving loss of muscle function and metabolic homeostasis. Gene ontology analysis of Affymetrix analysis demonstrated substantial loss of muscle specific genes, a global oxidative stress response related to most probably cytokine signalling, altered insulin related signalling and a substantial overlap between patients and muscle wasting/inflammatory animal models. MicroRNA 21 processing appeared defective suggesting that post-transcriptional protein synthesis regulation is altered by disruption of tissue microRNA expression. Finally, we were able to demonstrate that the phenotype of skeletal muscle in ICU patients is not merely one of inactivity, it appears to be an actively remodelling tissue, influenced by several mediators, all of which may be open to manipulation with the aim to improve clinical outcome.

**Conclusions/Significance:**

This first combined protein and transcriptome based analysis of human skeletal muscle obtained from septic patients demonstrated that losses of mitochondria and muscle mass are accompanied by sustained protein synthesis (anabolic process) while dysregulation of transcription programmes appears to fail to compensate for increased damage and proteolysis. Our analysis identified both validated and novel clinically tractable targets to manipulate these failing processes and pursuit of these could lead to new potential treatments.

## Introduction

Yearly about 750,000 people develop severe sepsis in the USA and probably the same amount in Europe. Total mortality for these patients is about 30–35%, however for those patients with persistent sepsis mortality is >50%. Most patients with persistent sepsis develop multiple organ failure, a syndrome in which several organ systems are malfunctioning. In order for these patients to survive their vital organs need to be supported in the hospitals intensive care unit (ICU). Septic patients treated in the intensive care unit develop skeletal muscle dysfunction which is part of the multiple organ failure syndrome, and this persists after ICU discharge [1], [2], [3], [4], [5]. The nature of this muscle dysfunction includes weakness due to a severe loss of muscle mass and muscle fatigue which is most apparent during weaning of the mechanical ventilation and results in impaired physical capacity during the patient's protracted recovery process [6]. In addition to the long term failure of skeletal muscle function, rapid degeneration in the ICU also impacts on patient acute energy metabolism and this directs the need for concurrent interventions, such as insulin and glucocorticoid therapy, which are principally aimed at improving patient survival [7]. In a previous study [8] we demonstrated that mitochondrial content was 30–40% lower and cellular adenine nucleotide homeostasis disrupted (lower ATP and creatine phosphate concentrations) in skeletal muscle of ICU patients suffering from sepsis induced multiple organ failure (MOF).

Mitochondria are the major mechanism for ATP generation in humans and the observed lower mitochondrial content and cellular energy status will accelerate muscle fatigue and possibly cell death in these septic patients [9], [10], [11]. Indeed, mitochondrial derangements and the subsequent disruption in energy metabolism are associated with multiple organ failure and an increased mortality in critically ill patients [12], [13], [14]. In addition, several animal models of sepsis and critical illness have shown mitochondrial derangements in skeletal muscle and other tissues [11], [15], [16], [17], [18] confirming the generality of these observations.

Skeletal muscle phenotype and mitochondrial content depend on the coordinated expression of nuclear and mitochondrial encoded genes, as well as the synthesis and degradation of proteins to maintain normal muscle function. Mitochondrial protein synthesis and degradation have to be in equilibrium in order for the cell to maintain a constant number of well functioning mitochondria. In this study we hypothesize that the lower mitochondrial content, we found in skeletal muscle of septic patients, is caused by a lower mitochondrial protein synthesis and this would be regulated by lower mitochondrial gene expression. Thus, we examined *in vivo* mitochondrial protein synthesis in skeletal muscle of patients treated in the ICU for sepsis induced MOF and compared this to age matched control subjects. Targeted analysis of gene expression of mitochondrial oxidative phosphorylation (OXPHOS) enzymes (both nuclear and mitochondrial encoded), mitochondrial proteases and master transcriptional regulators of mitochondrial biogenesis presented us with a complex picture, where selective transcriptional activation of mitochondrial biogenesis was clearly evident. Finding clear evidence for disrupted coordination of mitochondrial gene expression led us to carryout a global analysis of skeletal muscle phenotype using microarray technology to determine the extent of altered muscle phenotype. Informatic analysis yielded profound evidence for degeneration and loss of muscle specific genes.

## Materials and Methods

### Subjects

Seventeen patients admitted to the general Intensive Care Unit (ICU) at Karolinska University Hospital Huddinge were included in the study. The same ICU patients were also included in another study [19]. Patients were septic according to the Bone criteria [20]. Patients younger than 18 years of age, patients with severe liver failure, undergoing dialysis, and patients with impaired coagulation were excluded from the study. As a control group 10 patients undergoing elective surgery at Ersta Hospital (Stockholm) were included. All patients or close relatives gave informed consent to participate in the study. The study protocol conformed to the ethical guidelines of the 1975 declaration of Helsinki and had received an a priori approval by the Ethical committee of Karolinska Institutet, Stockholm, Sweden and local ethics approval for gene expression analysis at Heriot-Watt University, Edinburgh, Scotland.

### Study protocol

In order to determine the mitochondrial protein synthesis rates an intravenous bolus injection of L- [^2^H_5_] phenylalanine was given to the patients over a period of 10 minutes (45 mg×kg^−1^ body weight, 10 atom% excess (APE)) [21]. Blood samples were taken before (time 0) and at 5, 10, 15, 30, 50,70 and 90 minutes after the phenylalanine injection for determination of the L- [^2^H_5_] phenylalanine enrichment in plasma. Muscle biopsies were obtained from the lateral portion of the vastus lateralis muscle, 10–20 cm above the knee, at 90 and 92 minutes after the bolus injection on the left and right leg respectively. The biopsies were obtained using a Bergström biopsy needle after local anaesthesia in the ICU patients and just after sedation in the control patients. The biopsies were divided into smaller portions, frozen into liquid nitrogen and stored at −80°C until analysis.

The muscle biopsies were analyzed for *in vivo* mitochondrial protein synthesis rate, mitochondrial enzyme activities (citrate synthase and complex I and IV of the mitochondrial respiratory chain) and a marker of oxidative stress capacity (Superoxide dismutase (SOD)). In addition, mRNA levels were measured using real time qPCR and Affymetrix micro-array analysis. From our previous study it is clear that the between-patient variation of the mitochondrial enzyme measurements is high, therefore measurements in the present study were carried out in both legs to further examine this issue. For the mRNA analyses limited material was available and therefore only one leg was studied in 24 patients (16 septic and 8 controls) for the qPCR and 21 patients (13 septic and 8 controls) for the micro-array analysis.

### Measurements of enzyme activities

Muscle samples were homogenized in a KCL buffer (100 mM KCl, 50 mM Tris/HCl, 5 mM MgCl_2_, 1.8 mM ATP and 1 mM EDTA) to obtain a 5% homogenate [8], [22]. Part of the homogenate was stored frozen at −80°C. The remaining homogenate was used to isolate mitochondria by subsequent centrifugations [8], [22]. The mitochondrial pellet intended for enzyme activity measurements was suspended in SET buffer (0.25 M sucrose, 2 mM EDTA, 10 mM Tris, pH 7.4) and stored frozen until analysis at −80°C. Activities of citrate synthase, mitochondrial respiratory chain complexes I and IV and superoxide dismutase (SOD) were analyzed in both total homogenate and isolated mitochondria. All assays were spectrophotometric and performed on a Konelab 20 Analyzer (Thermo electron corporation, USA) at 37°C as described previously [8], [22]. SOD activity was analysed using a Ransod kit (Randox Laboratories Ltd, UK).

### Mitochondrial protein synthesis

Mitochondria were isolated as described above, but the mitochondrial pellet were dissolved and washed twice with 4% sulfosalicylic acid (SSA). The pellets were subsequently dissolved in 0.3 M NaOH and the protein concentrations of the samples were determined using a DC-protein assay kit (Bio-Rad Laboratories, Hercules, CA, USA). The proteins were then precipitated and washed twice in 4% (w/v) SSA. The remaining pellets were hydrolyzed at 110°C for 24 hours in 6 M HCl. After cooling, the samples were dried by vacuum centrifugation.

The dried samples were prepared for gas chromatography-mass spectrometry analysis as described previously [23]. The dried protein hydrolysates were dissolved in 220 µl of 0.5 M sodium citrate buffer (pH 6.3) and filtered through a 0.22 µm centrifugal tube. Phenylalanine standards containing 0–0.114 APE (atom percent excess) were dissolved in the same amount of the sodium citrate buffer. Samples and standards were decarboxylated in L-tyrosine decarboxylase to convert phenylalanine into phenylethylamine at 50°C over night. Subsequently, 100 µl of 6 M NaOH was added and after centrifugation at 16,000×g, the samples were extracted in ether and then in 0.1 M HCl and dried. The samples were derivatized in 25 µl MTBSTFA [N-methyl-N-(t-butyldimethylsilyl)trifluoroacetamide]/ethyl acetate(1/1, v/v) at 60°C for 1 hour. The ratio of [^2^H_5_]-phenylethylamine and phenylethylamine was determined by GC-MS (Agilent 5973n; Agilent technologies, Stockholm, Sweden). Ions m/z 180 (m+2) and m/z 183 (m+5) were analyzed. Plasma free phenylalanine was prepared and analyzed by GC-MS as described previously [21]. The enrichment of [^2^H_5_]-phenylalanine in plasma was determined by monitoring the ions *m/z* 336 and 341. The enrichment of protein-bound [^2^H_5_] phenylalanine was calculated from the ratio of m+5/m+2 using the standard curves. The protein synthesis rate was calculated as:
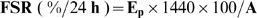
Where FSR is the fractional protein synthesis rate, E_p_ is the protein-bound enrichment of [^2^H_5_] phenylalanine (APE), A is the area under the curve for [^2^H_5_] phenylalanine enrichment (APE) in plasma over time.

### Quantitative real-time PCR

The transcript levels of several mitochondrial protein subunits encoded by either nuclear or mitochondrial DNA were measured by real-time (RT)-PCR. The nuclear encoded subunits of mitochondrial enzymes citrate synthase (CS var1 and 2), complex IV (COXIV) and succinate dehydrogenase (SDHA) were measured. For the mitochondrial-encoded genes, subunits of complex I (mtND4, mtND6) and complex IV (mtCO1) and cytochrome B (mtCYB) were measured. In addition, transcription factors NRF-1 and NRF-2α/GABP as well as coactivators PGC-1α and -1β were quantified. These transcriptional regulators control the expression of nuclear encoded mitochondrial proteins. To further examine factors which directly regulate mtDNA encoded genes, we determined TFAM, TFB1M and TFB2M expression. In addition, given our previous data, we quantified several mitochondrial protease genes, two that are part of the AAA proteases family and located in the inner membrane of the mitochondria (YME1L1 and SPG7/paraplegin) and two located in the mitochondrial matrix (LON and CLPP).

Total RNA was isolated from muscle samples using TRIzol (Invitrogen, Stockholm, Sweden) and quantified using a Nano-drop spectrophotometer. cDNA was prepared from 1 µg of RNA using random hexamer primers and reverse transcription reagents (Applied biosystems) in a final volume of 40 µl. Gene expression was quantified in triplicates as previously described [24]. Oligonucleotide primers were designed using a primer design centre (http://www.probelibrary.com/) and synthesized by Invitrogen (Stockholm, Sweden). To avoid amplification of nuclear DNA the primers were designed to amplify across exon-exon boundaries. Mitochondrial DNA contamination can not be excluded using this procedure and a ‘contamination test’ was therefore performed. Total RNA was utilized instead of cDNA using primers towards a mitochondrial-encoded gene (MTCO1) and the resulting amplification plot was used to indicate the amount of mtDNA contamination. The mtDNA contamination was negligible (less than 3%) and thus had no impact on the analysis. The delta C_T_ (cycle threshold) values, calculated based on correction to 18sRNA, were calculated for each sample and statistical analysis was applied to this raw data. The linear value of each patient was then compared to the mean of all the control values to get an estimate of the mean increase/decrease and the variation between the samples.

### Affymetrix Microarray

Hybridization, washing, staining and scanning of the arrays were performed according to manufacturer's instructions (Affymetrix, Inc. http://www.affymetrix.com/). The Array design can be found at https://www.affymetrix.com. We utilized the Affymetrix U133+2 array platform with RNA preparations. Data presented in the paper was quality controlled using the Microarray Suite software (MAS 5.0). All array data were normalized through implementations of the MAS5 algorithm, to a global scaling intensity of 100. As a mean to control the quality of the individual arrays, all arrays were examined using hierarchical clustering to identify outliers prior to statistical analysis in addition to the standard quality assessments including scaling factors and housekeeper 5′/3′ ratios. No array failed these standard quality assessment procedures although patient 9 was a clear ‘outlier’. Nevertheless, patient 9 did not influence the global gene expression analysis, while for the qPCr analysis (using a separate RNA/Analysis technology) patient 9 did demonstrate the highest expression value for NRF2 (See results) indicating that this patient's gene expression status was genuinely altered to a much greater extent.

### Array analysis strategies

We have previously demonstrated that it can be difficult to predict the impact of applying arbitrary filtering criteria prior to statistical analysis [25]. We therefore relied on several statistical models to present, analyze and interpret our data with and without arbitrary pre-filtering of gene lists. The microarray data was subjected to global normalization using the Robust Multi-Array Average expression measure (RMA) Bioconductor (www.bioconductor.org) and the output compared with MAS5.0. On this occasion 90% of RMA identified genes were found in the MAS5 data set while analysis of the smaller RMA data set provided a very similar result (in terms of Gene ontology analysis of differentially expressed genes). Thus, we used MAS5.0 for the remaining analysis as the point of array analysis is to generate a large yet robust data set. There is some argument that using the MAS5.0 generated present-absent calls can improve the sensitivity of the differential gene expression analysis [26]. We chose to remove probe sets where they were declared ‘absent’ across all chips. The normalized log2-file was analyzed with the Significance Analysis of Microarray (SAM) in R (http://www-stat.stanford.edu/~tibs/SAM/). SAM provides a list of “significant” genes and attempts to estimate of the false discovery rate (FDR), which represents the percentage of genes that could be identified by chance. SAM assign a score to each gene/probe set as an index of relative difference between groups. For the data presented in the manuscript, genes were considered significantly changed in ICU patients, when a delta value corresponding to the number of false significant genes of 5% (q-value), and an average fold change of 2, was achieved. For the pathway analysis a stricter statistical cut-off was utilized (1% FDR), while presented pathways were colored by 1.5 FC and 5% FDR so that the robust trends within pathways could be visualized. Finally, due to lack of sufficient RNA please note we did not profile the two patients with the very high protein synthesis rates on Affymetrix arrays and thus these patients did not drive nor inform the array analysis.

We used the web-based bioinformatics tool, Ingenuity pathway analysis (IPA, http://www.ingenuity.com), which is based on >1.7 Million published articles, to discover networks regulated in the skeletal muscle of ICU patients, compared with age matched controls. IPA is a knowledge database generated from the peer-reviewed scientific publications that enables discovery of biological networks in gene expression data, determining the functions most significant to those networks. Gene name identifiers or Affymetrix probe set ID's were uploaded into IPA and queried against all other genes stored in the IPA knowledge database. Each Affymetrix probe set ID was mapped to its corresponding gene identifier in the IPA knowledge database. Probe sets representing genes having direct interactions with genes in the IPA knowledge database are called “focus” genes, which were then used as a starting point for generating functional networks. Each generated network is assigned a score according to the number of differentially regulated focus genes in our dataset. These scores are derived from negative logarithm of the *P* indicative of the likelihood that focus genes found together in a network due to random chance. Scores of 14 or higher have 99.9% confidence level of significance. In reporting our findings we focus on networks with a substantially higher confidence limit and thus represent strong evidence for a given biological pathway being activated in ICU patients. It should be noted however that while the database extends the interpretation beyond mRNA transcript levels (as network genes don't have to be differentially expressed at the mRNA level) the database is finite and reflects current knowledge.

### Detection of microRNA hsa-mir-21 expression by using real-time PCR

MicroRNA expression was analysed using the Taqman® MicroRNA assays developed by Applied Biosystems that detects mature miRNA. A microRNA-specific looped primer is used for the reverse transcription, thus extending the microRNA sequence enabling detection with Taqman® real-time PCR. Samples were analysed for the expression of hsa-mir-21 (4373090, Applied Biosystems) and the endogenous reference, RNU48 (4373383, Applied Biosystems), which is a small nuclear RNA (which we have found to be stable in human muscle through comparison with 18s). Ten ng of total RNA was reverse transcribed using the TaqMan® MicroRNA Reverse Transcription Kit (Applied Biosystems, PN 4366597) according to instructions. In short, a mastermix was prepared resulting in a final concentration in a 15-µl volume of 1 mM dNTP, 50 U MuLV Multiscribe reverse transcriptase, 1× reverse transcription buffer and 3.8 U RNase Inhibitor. RNA was added, and aliquots made followed by addition of the microRNA-specific reverse transcription looped primer to a final concentration of 1× RT-primer. The reactions were gently mixed and briefly spun down and run in a PTC-100™(MJ Research Inc.) with conditions at 16°C for 30 min, 42°C for 30 min, and 85°C for 5 min.

For the real-time PCR, the TaqMan® 2× Universal PCR Master Mix, No AmpErase® UNG was used (Applied Biosystems, PN 4324020). The samples were run on a 7900HT Fast Real-Time PCR System (Applied Biosystems) on the 9600 emulation mode in triplicates of 20 µl per well. Real-time PCR conditions were; 95°C for 10 minutes followed by 50 cycles of 95°C for 15 sec and 60°C for 1 min. All reactions were run singleplex and analysed and quantified using the ΔΔCt method. Data are expressed as fold change from controls.

### miRNA target prediction and Gene Ontology Class

The binding of the miRNA to the target mRNA occurs in the “seed” region of the miRNA, representing nt 2–7 of the 5′end of the mature miRNA and the 3′ untranslated region (UTR) of the mRNA. We used Gene Ontology analysis to obtain an overview of the main classes of biological functions of genes predicted to be targets for miRNA-21 and thus we are able to apply a degree of statistical rigor beyond predicted target lists only. For miRNA target prediction categorized by Gene Ontology, we used EASE (Version 2 with updated annotation files, 2006) and a miRNA target gene list (representing target genes conserved across murine and human genes) developed based on Ensemble and miRNA sequences obtained from the Sanger Institute (http://microrna.sanger.ac.uk/sequences/). EASE analysis predicts biological functions of target genes for the miRNA of interest and enlists a false discovery rate (FDR) and an EASE score, which we calculated using 500 permutations/iterations. For prediction of miRNA-21 target sites we used Targetscan (http://www.targetscan.org/, release 4.0).

### Statistics

Most measurements were normally distributed according to the Kolmogorov-Smirnov test (Statistica, Softstat, Tulso, OK, USA). Since not all parameters were normally distributed we analysed all data (except the micro-array data) both using the Student's t-test and Mann-Whitney U-test. Data are presented as means and standard deviation. Statistical analysis for the micro-array is described in detail above.

## Results

### Included patients

ICU patient characteristics are given in Table 1. Despite their initial diagnosis all included patients were diagnosed with severe sepsis/septic shock [20] sometime during their disease. At the time of the biopsy, all patients were circulatory stable and not suffering from severe sepsis/septic shock. At the time of biopsy all patients were considered to suffer from multiple organ failure, although three patients had SOFA score below 4. All but one patient received intravenous insulin (20–260 IE per day of Actrapid, Novo Nordisk) to control hyperglycemia. Glucose levels were between 3 and 12 mM on the day of the study. As controls, 1 woman and 9 men were included, the median age was 70 (range 48–76) and median body mass index (BMI) was 27.4 kg×m^−1^. The control subjects underwent elective surgery for either gallbladder disease or inguinal hernia.

**Table 1:** Patient characteristics.

Diagnosis	Age/sex	ICU days*	Survival	SOFA	APACHE II**	Glucocorticoid	Nutrition***	Sedation	Vassopressor agents
Abdominal aortic aneurysm, pneumonia	60/F	2	S	7	12	Suplementation	EN	Propofol	yes
Pneumonia	47/M	1	S	9	19	None	PN	Propofol	yes
Stroke, pneumonia, cardiac arrest	63/M	42	D. day 82	8	19	Suplementation	PN+EN	None	no
Respiratory failure (surg)	72/F	1	S	5	22	Suplementation	EN	Propofol/midazolam	yes
Sepsis	77/M	2	S	1	27	Suplementation	EN	Propofol	yes
Respiratory failure, COPD	57/F	1	S	6	30	Replacement	PN+EN	Propofol	yes
Respiratory failure (surg)	68/M	2	S	2	25	None	PN+EN	Propofol	no
Eosofageal resection, COPD	77/M	7	S	5	21	None	PN	Propofol/midazolam/ketogan	yes
Respiratory failure, COPD	69/F	1	S	6	21	Replacement	EN	Propofol/midazolam/ketogan	yes
Pneumonia, COPD	77/M	3	S	5	39	Replacement	PN	Propofol	yes
Abdominal aortic aneurysm	73/F	35	D. day 95	4	16	Suplementation	PN+EN	Propofol/ketogan	yes
Multiple rib fracture	74/M	6	D. day 16	9	19	None	PN+EN	Propofol	yes
Respiratory failure and AMI	66/F	2	S	4	21	Replacement	PN+EN	Propofol	yes
Pneumonia	69/M	2	S	7	30	None	PN	Propofol	yes
Respiratory failure	48/M	2	S	7	14	None	PN	Propofol/ketogan	no
Abdominal sepsis (surg), COPD	71/F	1	D. day 13	12	29	Suplementation	PN	Propofol	yes
Abdominal sepsis (surg), COPD	25/F	6	S	3	13	None	PN+EN	Propofol	yes
Mean±SD	64±14	6.8±11.8		5.9±2.8	22.2±7.1				
Mean±SD for control subject	64±10								

SOFA; Sequential Organ Failure Assessment score, AMI; Acute Myocardial Infarction; COPD; Chronic Obstructive Pulmonary Disorder, PN; parenteral nutrition, EN; Enteral nutrition, S; Survivor, D, death, d; day, surg; surgery ^*^ Days spent in the ICU at the time of muscle biopsy ^**^ APACHE II score at admission to the ICU^***^ Nutrition was given at 20–25 kcal/kg/day including supplementation of glutamine.

### Muscle mitochondrial enzyme activities are decreased with sepsis

There were no significant differences between left and right legs in any of the enzyme and *in vivo* protein synthesis analyses such that all results are presented as the mean value obtained from two muscle biopsies. The activity of citrate synthase was 25% lower in the septic patients as compared to the controls (p = 0.0003; t-test) (Table 2). Mitochondrial respiratory chain complexes I and IV were respectively 49% and 33% lower in the patients when expressed per muscle weight (complex I p = 0.0002, complex IV p = 0.0003; t-test) (Table 2). However, no statistically significant difference was observed when expressed per citrate synthase activity (p = 0.10 and 0.13 respectively; t-test) suggesting that it was the total mitochondrial content that had declined. In isolated mitochondria, the activity of complex I was not statistically significantly different between controls and patients (p = 0.11; t-test), while intriguingly the activity of complex IV was 60% greater in the patients (p = 0.0003; t-test) (Table 2). Superoxide dismutase activity in muscle homogenate was not different between the patients and the controls (p = 0.79; t-test), yet the activity in isolated mitochondria was 100% higher in the patients (p = 0.011; t-test) (Table 2). We carried out protein and gene expression analysis to investigate the cause of these alterations in enzyme capacity.

**Table 2:** Enzyme activities.

	ICU (n = 17)	Controls (n = 10)
**Citrate synthase** muscle (µmol×min^−1^×g^−1^ ww)	18.7±4.3*	24.3±4.6
**Complex I** muscle (µmol×min^−1^×g^−1^ ww)	1.9±0.8 *	2.9±1.0
**Complex I** muscle (U×U^−1^ CS)	0.10±0.03	0.12±0.04
**Complex I** isolated mitochondria (U×U^−1^ CS)	0.41±0.12	0.35±0.06
**Complex IV** muscle (µmol×min^−1^×g^−1^ ww)	8.1±2.6*	11.5±2.9
**Complex IV** muscle (U×U^−1^ CS)	0.43±0.07	0.46±0.09
**Complex IV** isolated mitochondria (U×U^−1^ CS)	1.42±0.27*	0.94±0.21
**Total SOD** (U×g^−1^ ww)	217±30	220±17
**Mitochondrial SOD** (U×U^−1^ CS)	1.25±0.85*	0.50±0.15

Mean and standard deviation of mitochondrial enzyme activity measurements in leg muscle from ICU patients with sepsis induced multiple organ failure. ^*^ p<0.05 (both for Student's t-test and Mann Whitney U-test). ICU intensive care unit patient, ww wet weight, CS citrate synthase, SOD superoxide dismutase

### The decreased mitochondrial function is not due to a decreased in vivo protein synthesis rate

**Figure 1:**
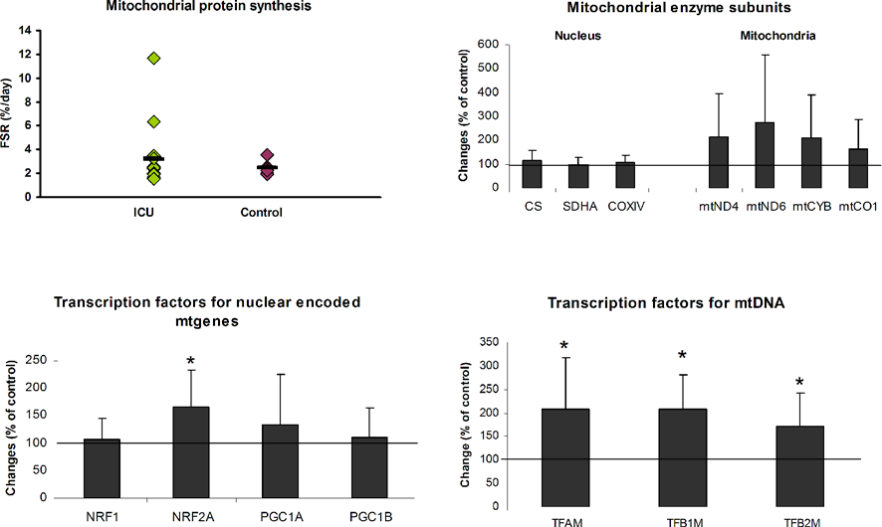
Protein synthesis and gene expression of mitochondria in muscle of septic and age-matched control subjects. *In vivo* fractional synthesis rate (FSR) of mitochondrial protein in skeletal muscle from ICU patients with sepsis induced multiple organ failure and age-matched control subjects are expressed in %/day (top left panel). Each dot represents one patient and the bar represents the mean value. mRNA level of subunits of nuclear and mitochondrial encoded enzymes (top right panel), transcription factors controlling expression of nuclear encoded proteins (lower left panel) and transcription factors regulating the transcription of mitochondrial DNA (lower right panel) in skeletal muscle of septic patients. The changes in mRNA levels are expressed in percent of controls (n = 8) and are presented as mean and standard deviation. The genes marked with mt are mitochondrial encoded. *p<0.05 (both for Student's t-test and Mann-Whitney U-test).

The amount of mitochondrial protein and therefore muscle mitochondrial content is the balance between mitochondrial synthesis and breakdown. To establish whether the decreased mitochondrial function in muscle of septic patients is due to a decreased synthesis of mitochondrial proteins, *in vivo* protein synthesis rates of mitochondrial protein was determined. No significant difference in mitochondrial protein synthesis was obtained between the patients and controls (p = 0.35; t-test) (Figure 1A) albeit the patients did demonstrate a greater variability in synthesis rates, where some appeared to have a substantially elevated fractional synthesis rate.

### The decreased mitochondrial function may reflect incomplete mitochondrial biogenesis and increased expression of mitochondrial protease genes

To specifically address whether transcriptional activation of mitochondrial related genes was suppressed we utilised real time qPCR. None of the mitochondrial and nuclear genes, encoding for mitochondrial related enzymes were significantly different between patients and controls (Figure 1B). Further, while there was greater variation for the mtDNA-encoded genes than for the nuclear encoded oxidative phosphorylation (OXPHOS) genes the tendency was for up regulation. Assembly of mitochondria networks involves hundreds of gene products and while OXPHOS related mRNA expression may have been stable in patients, disruption of regulators of mitochondrial organelle/network formation could also result in loss of function. Surprisingly, the proposed master regulators of mitochondrial biogenesis; NRF1, PGC1α and PGC1β were unchanged, however NRF2α/GABP expression was 67% higher in the septic patients (p = 0.0163; t-test) (Figure 1C). Convincingly, the mtDNA regulating factors TFAM, TFB1M and TFB2M were all increased significantly (by 109, 108 and 72% respectively) and these nuclear encoded genes are regulated by NRF2α/GABP (Figure 1D).

Thus while we clearly demonstrated decreased mitochondrial enzyme activities, this was not due to lack of mRNA coding for OXPHOS proteins. Rather, increased expression of several of the transcriptional regulators of biogenesis suggests that there was a clear yet partially effective stimulus for mitochondrial biogenesis. This did not, however, result in an increased synthesis of mitochondria but rather only selective activation of NRF2α/GABP and NRF2α/GABP target genes. However, the ICU patients were treated with insulin therapy to overcome hyperglycemia [7] and experimental hyperinsulinaemia has been shown to increase the expression of some oxidative phosphorylation genes [27]. It therefore occurred to us that the insulin therapy may be responsible for alterations in NRF2α/GABP and that such changes were not specific to MOF. We analysed the response of NRF2α/GABP to exogenous insulin *in vivo* in humans and using a muscle cell line model. First we examined the expression of NRF2α/GABP in the C2C12 muscle cell line, exposed to supra-physiological levels of insulin and we could not detect any induction of NRF2α/GABP expression or its target genes TFB1M and TFB2M (Supplemental Figure S1), suggesting that increased NRF2α/GABP and NRF2α/GABP target gene expression in the ICU patients was not a consequence of the insulin therapy but rather some more specific response to altered mitochondrial function. We also observed a reduction in PGC1α mRNA in insulin treated C2C12 cells, consistent with the *in vivo* study by Southgate et al [28]. In addition, to verify that insulin would not increase NRF2α/GABP *in vivo* we utilised RNA derived from a previously published [29] hyperinsulineamia clamp study in healthy volunteers (5 hrs, 149.2+/−6.9 mIU×l^−1^) and we found that NRF2α/GABP and PGC1α mRNA expression was unaltered compared with control (data not shown). It appears unlikely that the robust activation of NRF2α/GABP and target genes in ICU patients reflects the insulin therapy. Connectivity analysis was carried out using NRF2α (GABPA and GABPB) and Affymetrix data (See below) further illustrates the response of some NRF2α/GABP related genes (Supplemental Figure S2). We also evaluated if mitochondrial located proteases were activated. Genes encoding for the mitochondrial proteases LON and CLPP demonstrated an increase in the patients compared to controls of 48 and 39% respectively (P = 0.0029 and 0.0166 respectively; t-test) while the AAA metalloprotease subunits paraplegin (SPG7) and YME1L1 genes did not differ between the patients and controls. These results support the idea that in muscle of patients with sepsis, deregulated mitochondrial biogenesis may promote mitochondrial proteases expression to cope with failure in coordinated protein formation. However, the substantial loss of skeletal muscle mitochondrial content appears disproportionate compared with the global yet incomplete activation of biogenesis (See below) and thus it was important to identify the molecular signals coordinating these gross alterations in muscle phenotype.

### Affymetrix analysis of ICU patient skeletal muscle shows a large number of genes that are differentially expressed, with the majority up regulated

To establish a more global analysis of muscle phenotype we subjected the muscle samples to Affymetrix gene array analysis followed by our comparative array analysis strategy [30], [31]. The data discussed in this publication have been deposited in NCBI's Gene Expression Omnibus and are accessible through GEO Series accession number GSE13205 (http://www.ncbi.nlm.nih.gov/geo/query/acc.cgi?accGSE13205). The micro-array analyses were initially used to obtain a more global picture of the mitochondrial genes in skeletal muscle during sepsis. In addition, this allowed us to generate a detailed picture of ICU patient muscle status and also facilitated a direct comparison with various animal models which have been developed to study muscle wasting and inflammation [32], [33] to aid future drug discovery efforts. This later analysis allows us to infer associations between changes in patient muscle with specific programmes activated by muscle wasting or muscle disuse - data that would otherwise not be possible to generate, given the critical status of such individuals. This enormously valuable analysis should also help further validate these animal models for drug target validation purposes.

We found that 2080 probe sets (corresponding to 1457 unique genes/identifiers) were up-regulated in ICU patients, compared with control subjects (>2 fold change with a 5% FDR (Supplemental data sheet S1). We found fewer down regulated genes, with 783 significant probe sets representing 525 unique genes/identifiers (>2 fold change with a 5% FDR). Examination of the Affymetrix expression data allowed us to take a more global view on the regulation of *mitochondrial* gene expression. From a list of 342 genes annotated as being ‘mitochondrial’ on the U133+2 micro-array chip, expression of 82 genes were regulated in muscle of the septic patients (Supplemental data sheet S2). Of these 82 genes, only 8 were down regulated and remarkably 74 were up regulated. From the down regulated genes only one was a specific component of the OXPHOS machinery (NADH dehydrogenase 1 subunit C1). Within the up regulated genes, the largest change observed was for the mitochondrial superoxide dismutase gene, with a 16-fold increased expression. This result is remarkably consistent with the increased activity of the mitochondrial superoxide dismutase protein activity (Table 2). Thus analysis of the mitochondrial gene responses, as detected using the Affymetrix platform, strongly supports our conclusion that activation of many but not all mitochondrial genes occurred; a potentially adaptive, yet clearly ineffective, reponse at maintaining mitochondrial capacity.

### Gene ontology analysis of Affymetrix analysis identifies broad molecular programmes activated in ICU patient skeletal muscle

To capture a broad assessment of the molecular processes represented by the ∼2000 modulated genes, we utilised gene ontology (GO) analysis [34]. We found that regulation of apoptosis, proteosome function, ion homeostasis and kinase signalling were modulated and these appeared to us as rather predictable findings (Supplemental data sheet S1). Gene ontology analysis of the down regulated list indicated that there was a dramatic loss of unique muscle related gene expression demonstrating that the patient muscle tissue was undergoing a de-differentiation process. Down regulated genes also indicated a loss of expression of numerous extracellular matrix gene ontology groups, which are known to be central to skeletal muscle remodelling and gain in physiological capacity [30], [35]. Thus, despite the lack of change in global protein synthesis, the types of protein being synthesised are most probably extremely different in the skeletal muscle of ICU patients.

As we wish to intervene to prevent the loss of muscle tissue function, it is important to determine which biological pathways were regulating this shift in muscle phenotype. To examine this question, we took a number of approaches. Using a multiple array analysis strategy [30], [31] we utilised the extensive animal models of muscle wasting, inactivity and inflammation from the Goldberg laboratory [32], [33]. While it is implausible to directly assess muscle function in the ICU setting, this comparative analysis allows us to contrast ICU patients with models of muscle atrophy, muscle inflammatory and muscle inactivity along the lines presented by Sacheck et al [33]. It also facilitates discussion of the utility of such models and whether they accurately represent human muscle wasting disorders. Secondly, we utilised Ingenuity pathway analysis to establish which signalling processes or unique gene-networks may be coordinating the altered tissue phenotype (Ingenuity IPA, http://www.ingenuity.com).

### Altered atrogen expression, described in catabolic animal models, are similar in the septic patients, except for the energy metabolism genes

In two recent publications, common genes up regulated or down regulated in animal models with muscle wasting have been identified [32], [33]. In the first study animal models for fasting, cancer cachexia, uremia and diabetes mellitus identified 120 unique genes involved in catabolism, and the authors named these ‘atrogens’. In a second paper these genes were compared with two animal models of disuse induced muscle wasting, leading to 53 commonly changed genes. We compared these results to our data from MOF patients and found good agreement, with the exception of the genes involved in energy metabolism (Supplemental data sheet S3). As described above, the majority of mitochondrial genes involved in energy production are either unchanged in muscle of the septic patients or modestly increased, and this agrees with our previous conclusions that in humans, mitochondrial related mRNAs are not highly responsive to alterations in muscle usage in humans [24], [30].

When comparing the septic patient responses in gene expression, with animal models of disuse, in general a much poorer agreement is observed. This indicates that muscle inactivity, inevitable in the septic ICU patients, doesn't dominate the muscle phenotype seen in these patients, while the *in vivo* disuse phenotype observed in the various model systems is highly reproducible. Pathway analysis revealed that the animal model atrogens formed several networks, of which estradiol was highlighted as a molecular regulator (Supplemental Figure S3). In support of this novel re-analysis of the data from the Goldberg laboratory, it has been demonstrated that estrogen status regulates muscle recovery from atrophy [36] and in the ICU patients ERS1 (estrogen receptor 1) was 3-fold down regulated. Notably estrogen receptors are present also in male skeletal muscle [37] and are proposed to attenuate atrophy [38]. Based on our analysis of the Goldberg laboratory models, and the fact that our patients present a similar muscle gene expression profile, interventions which promote muscle estrogen receptor activation may be worth investigating in the ICU setting.

### Pathway analysis identifies modulation of key canonical pathways and networks in MOF ICU patients

**Figure 2:**
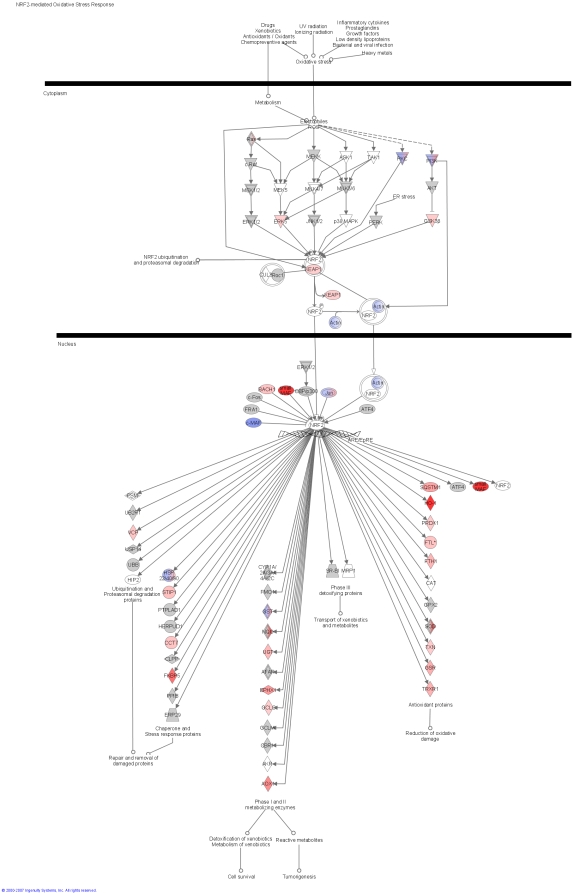
Nuclear Factor Erythroid 2 canonical pathway. Ingenuity analyses of micro-array data indicated a canonical pathway around nuclear Factor Erythroid 2 (Nrf2) as one of the most significantly (p = 1.4×10-5) changed in muscle of the septic patients in comparison with the age-matched controls. Gene descriptions can be found in supplemental data sheet S5. Red (and pink) means significantly increased gene expression and blue decreased. The ‘strength’ of the colour is an indication of how up regulated the gene was (with darker red being more up-regulated). Grey is a highly connected gene (to the transcript network that is altered) but itself is not altered at the mRNA level. Blue/Red hybrids are genes identified in the data base where the protein is actually at least two different genes and 1 sub-unit is down, while the other is up.

We found several significantly modulated canonical signalling pathways which understandably linked with sepsis. Nuclear Factor Erythroid 2-like-2 is a transcription factor (Nrf2, not to be confused with mitochondrial related gene NRF2α/GABP), which responds to a variety of mediators including inflammation. The Nrf2 mediated oxidative stress response pathway was highly regulated in ICU patients (Figure 2, p = 1.4×10-5 and supplemental data set S4) and recently it was shown that in Nrf2 -/- mice, mortality and inflammation is greatly enhanced in response to sepsis [39]. Nrf2 appears important for the regulation of glutathione synthesis and cellular detoxification processes. Restoration of glutathione levels in muscle of septic patients seems to be a prioritised system [40] that could be regulated via Nrf2. Our data suggests that Nrf2 plays a major role in muscle tissue during sepsis, perhaps coordinating the oxidative stress response.

**Figure 3:**
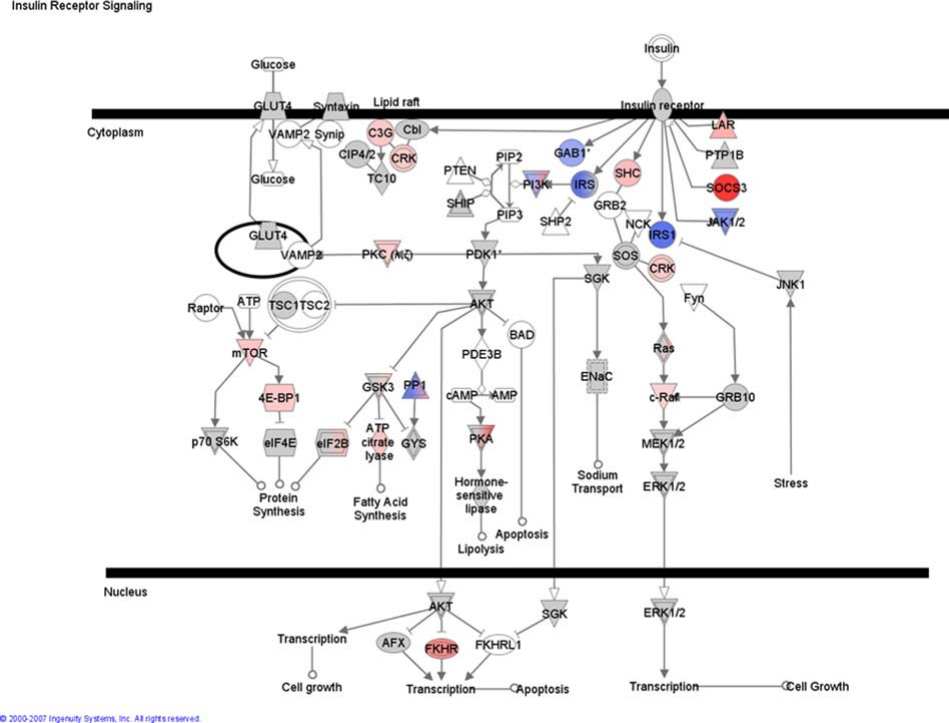
Insulin signalling canonical pathway. Ingenuity analyses of micro-array data indicated a canonical pathway involving insulin signalling as significantly changed in muscle of the septic patients in comparison with age-matched controls. Gene descriptions can be found in supplemental data sheet S5. Red (and pink) means significantly increased gene expression and blue decreased. The ‘strength’ of the colour is an indication of how up regulated the gene was (with darker red being more up-regulated). Grey is a highly connected gene (to the transcript network that is altered) but itself is not altered at the mRNA level. Blue/Red hybrids are genes identified in the data base where the protein is actually at least two different genes and 1 sub-unit is down, while the other is up.

We found that insulin receptor signalling pathway was significantly modulated in the patients (Figure 3 and supplemental data set S4). Given the critical role of insulin resistance in these patients [41] it was informative in that molecules immediately down stream of the insulin receptor appeared to be down regulated, such as IRS1, CD36 and JAK1/2 (a tyrosine kinase associated with cytokine and metabolic signalling). The exception was suppressor of cytokine signalling 3 (SOC3) which was 7 fold increased in expression (Figure 3 and supplemental data set S4) and this would be consistent with attempts to suppress the deleterious impact of MOF induced cytokine signalling [42] on insulin action as SOC3 can oppose inflammatory IL6 signalling. More distal insulin targets, such as mTOR and hormone sensitive lipase were up-regulated. These responses would suggest to us that the exogenous insulin interacts with excessive cytokine signalling to influence skeletal muscle gene expression. Additional canonical systems appeared activated such as JAK/Stat signalling and PPARα/RXRα. With respect to the later, AMPK and adiponectin expression was both elevated (both also associated with aspects of mitochondrial biogenesis), while in the same PPARα/RXRα influenced signalling system, down regulation of Stat5b indicated a potentially negative interaction between growth hormone action on muscle and PPARα. Activation of PPARα by using an agonist has been shown to improve mitochondrial content and function in skeletal muscle of children with severe burn injury [43], [44] providing a second example of where our data directs us to a drug target.

Finally, Gene Network analysis (based on gene expression changes and nearest molecular neighbours) demonstrated a large number of regulated gene expression networks in skeletal muscle of septic patients. The nuclear phosphoprotein MYC (V-MYC avian myelocytomatosis viral oncogene homolog) was 9 fold induced and was central to the highest scored network that included induction of 3 subunits of eukaryotic translation initiation factor 3 (Supplemental figure S4 and supplemental data set S4). Additional significant networks included ESR1 (down regulated) and genes associated with the atrogen analysis and TGFβ connected genes known to be important for muscle tissue remodelling (Supplemental figure S5, supplemental data set S4).

### Protein synthesis and altered processing of microRNAs

**Figure 4:**
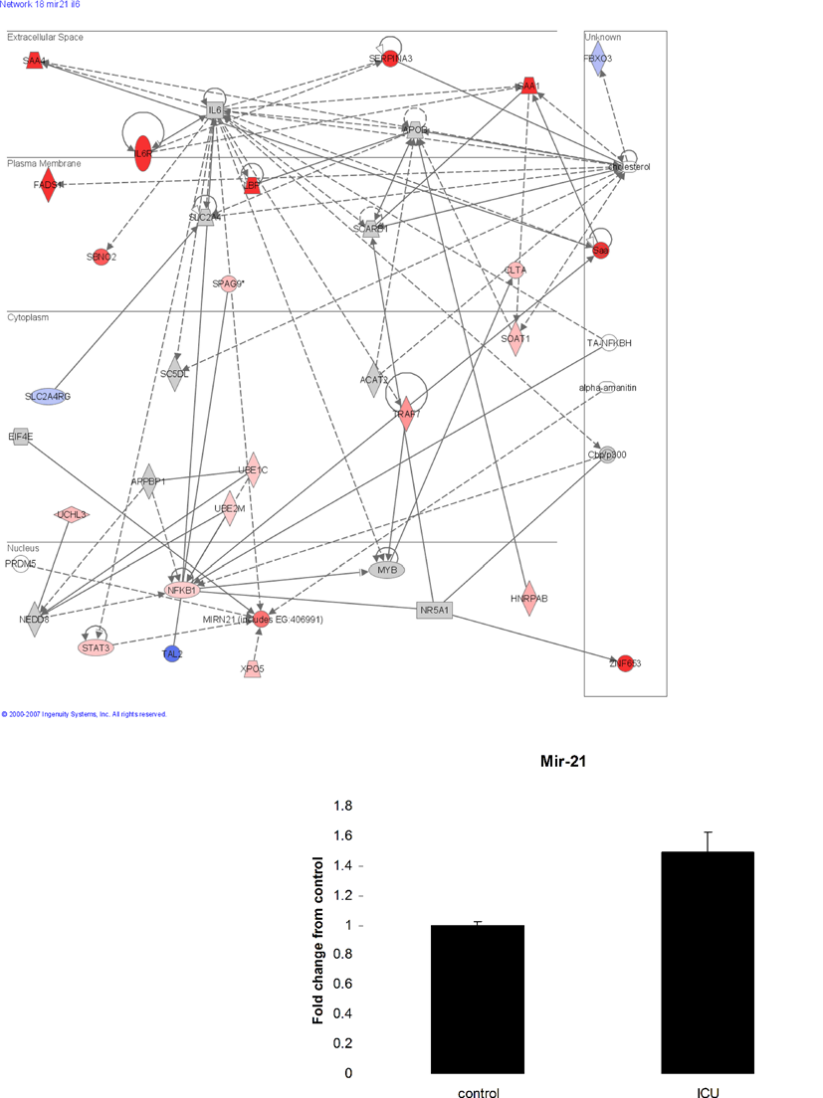
Interleukin- 6 and micoRNA –mir 21- related network. Ingenuity analysis indicated a network including interleukin-6 and the microRNA mir21 to have many significantly changed genes in the septic patients compared with the controls. Data in the micro-array is of pre-microRNA of mir-21 where as the qPCR data (insert) is of the mature and active microRNA. Data in the insert are fold change in mir-21 in septic patients compared with controls and are presented as means±SEM. Gene descriptions can be found in supplemental data sheet S4. Red (and pink) means significantly increased gene expression and blue decreased. The ‘strength’ of the colour is an indication of how up regulated the gene was (with darker red being more up-regulated). Grey is a highly connected gene (to the transcript network that is altered) but itself is not altered at the mRNA level. Blue/Red hybrids are genes identified in the data base where the protein is actually at least two different genes and 1 sub-unit is down, while the other is up.

Interestingly, given that global protein synthesis rates were unchanged in the patients, 27 translation related genes were up-regulated (e.g. Supplemental figure S4), while perhaps critically this up-regulated functional family did not include the rate-limiting translation initiation 5′ cap-binding protein, EIF4E [45]. Unchanged expression of EIF4E was found in an IL-6 influenced network demonstrating evidence for vastly up regulated IL-6 receptor and STAT3 (Fig. 4 and supplemental data set S4). Intriguingly, this network analysis of the Affymetrix array also included a 5-fold increase of the pri-microRNA transcript of mir-21 in ICU patients and microRNAs are selective regulators of protein synthesis [46]. In addition, XPO5, the exportin responsible for transporting the mature miRNA from the nucleus, was also increased substantially in the patients. We therefore went on to analyse the abundance of the mature and active microRNA, mir-21 in ICU patients and controls using real time qPCR method. The ICU patients displayed a variable and non-significant 50% increase in the mature mir-21 expression levels (p = 0.13), see Figure 4 insert, contrasting with the primary transcript. This suggests to us that suppressed microRNA processing or export may be occurring, representing a plausible mechanism explaining global loss of coordinated muscle gene expression and thus worthy of further investigation. This is especially plausible as Gene ontology analysis of the predicted targets of mir-21 suggest it should inhibit genes involved in ubiquitin ligase and JAK-STAT activity, both which were processes shown to be significantly up regulated in the transcriptomes of the patients. Thus failure to produce sufficient mir-21 may well have contributed to the activation of these pathways.

## Discussion

In this study we investigated the nature of the mitochondrial dysfunction found in intensive care using enzymology, protein flux analysis and transcriptomics. While some mitochondrial enzymes activities were 25–49% lower in skeletal muscle of patients treated in the ICU for sepsis induced multiple organ failure in comparison with a control group of similar age it was abundantly clear that this was not due to either a global reduction in mitochondrial gene transcripts or to impaired *in vivo* total mitochondrial protein synthesis. Our initial analysis demonstrated a selective activation of NRF2α/GABP and its target genes suggesting partial activation of mitochondrial biogenesis, while global analysis of 342 mitochondrial genes supported this interpretation. Comparative array analysis discovered that while many atrophy and inflammation responses are conserved across species, the metabolic gene responses in rodent models do not represent a response seen in ICU patients. This is particularly important, as gene expression profiling without consideration of physiological context can produce misleading conclusions [30]. Finally, network analysis was able to pin-point oxidative stress related pathway activation, modulation of the insulin signalling pathway and indicated that skeletal muscle in ICU patients was undergoing a de-differentiation process regardless of duration in the ICU.

### Metabolic homeostasis and mitochondrial dynamics

In our previous study, as well as in the present one, we found low activities of mitochondrial respiratory chain complexes I and IV, and citrate synthase when expressed per muscle weight. However, the low activities of complexes I and IV were not present when expressed per citrate synthase activity, indicating a net decrease in mitochondrial content had occurred. Low activities of mitochondrial enzymes in skeletal muscle have been shown before in animal models for sepsis [11], [15], [16], [17], [18] and in critically ill patients [12], [14], [47], [48]. Most patient studies included patients in the acute septic or cardiogenic shock [14], [47], [48] whereas our patients have been stabilized in the ICU and have developed multiple organ failure. During the acute critical phase results indicate a decreased function of the respiratory chain enzymes whereas the later phase of sepsis is characterized by a general decrease in mitochondrial content [49], [50]. One might assume that the loss of mitochondrial content reflects muscle inactivity resulting in decreased gene activation [24] or sepsis induced atrophy disrupting mitochondrial biogenesis [32]. However, neither appears to be true. Firstly, the *in vivo* mitochondrial protein synthesis in the septic ICU patients was not different from that in the control subjects, indicating that sufficient mRNA template existed for translation. Secondly, targeted qPCR analysis demonstrated that the expression of several nuclear encoded oxidative phosphorylation genes did not differ between the groups, while there was a trend for up-regulation of mtDNA encoded oxidative phosphorylation genes and global analysis identified >80 modestly up regulated nuclear encoded mitochondrial related genes. Critically, this pattern of gene expression differs markedly from muscle unloading in humans [24] and from animal models of atrophy-sepsis, indicating that our findings are not driven by inactivity nor are they adequately represented by preclinical models. These results indicate for the first time, that the observed lower mitochondrial content in muscle of septic ICU patients with multiple organ failure is not due to a general decrease in mitochondrial gene expression or failure in the protein synthesis process.

The balance between the biogenesis pathways and the rate of mitochondrial protein degradation regulates tissue mitochondrial content. The coordinate gene expression for the estimated 1500 or so mitochondrial proteins, is under tight control by these different transcriptional regulators [51], [52], [53], [54], [55], [56], [57], [58]. The nuclear encoded mitochondrial proteins are regulated by NRF1, NRF2α/GABP as well as PGC1α and β, while mitochondrial DNA replication and transcription is greatly influenced by TFAM, TFB1M and TFB2M [56], [58]. In the present study we found high mRNA levels of all three transcriptional regulators that act on mitochondrial DNA, and intriguingly selectively high NRF2α/GABP levels, a transcription factor that regulates the nuclear encoded TFAM; TFB1M and TFB2M. We also determined that the selective activation of NRF2α/GABP was unlikely to be caused by exogenous insulin. The inability of mitochondrial protein synthesis to sustain mitochondrial capacity may therefore reflect a lack of coordinated expression of all the transcription factors needed for an increased mitochondrial biogenesis. The reason for the lack of coordinated increase in mitochondrial biogenesis genes in these patients needs to be elucidated and may ultimately be manipulated to improve patient outcome.

### Regulation of protein synthesis and potential alterations in microRNA processing

It has previously been reported that mitochondrial protein synthesis and gene expression are decreased in septic rats [11], [59]. Like our present comparative analysis, this supports the idea that rodent models may be inappropriate for studying mitochondrial dynamics. The unchanged mitochondrial protein synthesis rates are in line with the synthesis rates of other muscle proteins in the septic ICU patients. The very characteristic loss of total muscle protein in these patients is also accompanied by normal synthesis rates of total muscle protein [19] while our transcriptomics indicates that the actual proteins being made, will clearly be substantially different from control skeletal muscle. Future detailed proteomic analyses combined with ribosomal RNA analysis [60] will allow us to define which proteins are now being synthesised.

The translational initiation factors (EIFs) are central components of the protein translation machinery and EIF4 is the proposed rate limiting factor for protein translational. While the partners of EIF4E, EIF4A1 (Helicase), EIF4G1 (scaffold protein) and EIF3S10 were all up-regulated in the ICU patients, EIF4E is unchanged at the mRNA level. In combination with the increased expression of the tRNA synthetases, MARS, LARS and RARS it would appear that a substantial yet incomplete attempt was made to promote muscle protein synthesis, similar to that observed for the mitochondria. This appears to have failed perhaps reflecting a lack of up-regulation of EIF4E, however, EIF4E is not considered to be regulated to a great extent, at the mRNA level [61], and thus further analysis is merited. Overall, it is clear that the lack of gross changes in *in vivo* protein dynamics must obscure critical alterations in specific protein formation, such that combing protein measurements with global transcriptomics provides a powerful solution to understanding the nature of tissue remodelling in multiple organ failure patients.

When looking for novel regulators of altered protein production from mRNA, microRNA's as global regulators of protein synthesis are of great interest [62]. In the ICU patients, we found a compelling 500% increase in the precursor for mir-21 (pri-mir-21). When we measured the mature form of mir-21, we observed no significant change in mir-21 levels (50% increase, p = 0.13) in skeletal muscle of ICU patients when compared with controls. This is important as it has been recently identified that reduction in PTEN expression could be a compensatory mechanism to prevent muscle protein degradation [63] and mir-21 is a validated target of PTEN [64]. Mature mir-21 is processed from a 3,433 long nucleotide pri-mir-21 [65]. Thus, despite the substantial increase in the mir-21 precursor, only modest changes in mature miRNA occurred.

Interestingly, IL-6 is activated in ICU patients [42] and has been reported to increase mir-21 in tumour cell lines mediated via STAT3 [66]. The IL-6 receptor increases in skeletal muscle in response to both acute [67] and chronic [68] stimuli, suggesting an increased responsiveness to IL-6. We detected a 10-fold increase in the IL-6 receptor in skeletal muscle of ICU patients, when compared with healthy controls, along with a 1.8-fold increase in STAT-3, the intracellular mediator of IL-6 signalling. This suggests an increased sensitivity to IL-6 and an increased IL-6 response in the ICU patients and this may explain the substantial increase in transcription of the precursor for mir-21. Whether global processing of microRNA's has been disrupted in ICU patients merits further analysis. It would appear, from this initial analysis, that there is substantial transcriptional drive to promote mir-21 expression, while some aspect of the miRNA biogenesis pathway limits substantial production of this translational blocking RNA species.

### Clinical impact for the patient

The loss of muscle mass and function has a profound affect on mortality and morbidity, not only in the septic ICU patients included in this study but also in other diseases with more chronic muscle wasting e.g. cancer, kidney failure and COPD. In the septic patients the loss of muscle mass as well as mitochondrial content are not due to an overall decrease in their synthesis rates. A obvious conclusion from this is that the losses are driven by increased degradation and that therapeutic aims should be focussed on modulating inappropriate degradation. However the present data suggest that a different spectrum of protein will be synthesised in these patients, and this implies that the precise profile of proteins being synthesised is altered and thus targeting protein degradation is not necessarily a logical strategy.

### Limitations of the study

A potential limitation of the present study is the heterogeneity of the included patients. Patients were included at different days of ICU treatment (range from 1–42 days). It is possible that the mitochondrial derangements are different at different phases of disease. However, it is not certain that the duration of ICU stay represents different phases of the disease. Often patients treated in the ICU have been ill for a while before, whereas others arrive to the ICU early after the initial insult. It is therefore difficult to see length of ICU stay as a measure of time of disease. However when the patient with a shorter ICU stay (<6 days) were compared with those with a longer stay (≥6 days) no significant difference were observed (Table 3 and 4). Also post-hoc analyses not including the 2 patients with extreme long ICU treatment (35 and 42 days) showed exactly the same changes in comparison with the controls as when including all patients (Table 3 and 4). Most importantly, global transcript profiling and clustering indicates that all patients are remarkably similar and develop a specific and clear muscle phenotype.

**Table 3:** Post hoc analyses on enzyme activities and mitochondrial synthesis rates.

	<6 days ICU (n = 12)	>6 days ICU (n = 5)	p =
**Citrate synthase** muscle (µmol×min^−1^×g^−1^ ww)	19.5±3.2	17.1±6.1	0.31
**Complex I** muscle (µmol×min^−1^×g^−1^ ww)	1.9±1.6	1.6±1.1	0.57
**Complex IV** muscle (µmol×min^−1^×g^−1^ ww)	8.5±2.0	7.2±4.0	0.35
**Total SOD** (U×g^−1^ ww)	212±20	224±42	0.43
**Mitochondrial SOD** (U×U^−1^ CS)	0.9±0.4	1.6±1.3	0.15
**Synthesis rates** (%/day)	2.3±0.6	4.6±3.9	0.05
	**<7 days ICU (n = 15)**	**Controls (n = 10)**	**p = **
**Citrate synthase** muscle (µmol×min^−1^×g^−1^ ww)	19.4±4.1	24.3±4.6	*0.01*
**Complex I** muscle (µmol×min^−1^×g^−1^ ww)	1.9±0.8	2.9±1.0	*0.01*
**Complex IV** muscle (µmol×min^−1^×g^−1^ ww)	8.6±2.5	11.5±2.9	*0.01*
**Total SOD** (U×g^−1^ ww)	217±27	220±17	0.71
**Mitochondrial SOD** (U×U^−1^ CS)	1.1±0.8	0.5±0.1	*0.03*
**Synthesis rates** (%/day)	2.4±0.6	2.5±0.4	0.91
	**ICU SOFA>3 (n = 14)**	**Controls (n = 10)**	**p = **
**Citrate synthase** muscle (µmol×min^−1^×g^−1^ ww)	18.8±4.1	24.3±4.6	*0.006*
**Complex I** muscle (µmol×min^−1^×g^−1^ ww)	1.8±0.7	2.9±1.0	*0.007*
**Complex IV** muscle (µmol×min^−1^×g^−1^ ww)	8.1±2.4	11.5±2.9	*0.005*
**Total SOD** (U×g^−1^ ww)	212±27	220±17	0.39
**Mitochondrial SOD** (U×U^−1^ CS)	1.1±0.6	0.5±0.1	*0.007*
**Synthesis rates** (%/day)	3.4±2.7	2.5±0.4	0.30

Data is given as mean and standard deviation. p for Student's t-test. ICU intensive care unit patient, ww wet weight, CS citrate synthase, SOD superoxide dismutase

**Table 4:** Post hoc analyses on mRNA levels measured with qPCR expressed as percentage of control.

	<7 days ICU (n = 14)	P =	ICU SOFA>3 (n = 14)	p =
**CS**	122±40	0.40	124±36	0.14
**SDHA**	93±32	0.49	100±27	0.98
**COXIV**	103±31	0.93	108±27	0.53
**mtND4**	218±176	0.06	211±177	0.06
**mtND6**	278±268	0.05	250±272	0.18
**mtCYB**	220±176	0.36	209±180	0.46
**Mt CO1**	171±123	0.51	165±124	0.55
**NRF1**	104±37	0.84	107±35	0.67
**NRF2/GABP**	169±68	*0.02*	175±63	*0.005*
**PGC1A**	121±85	0.95	136±80	0.23
**PGC1B**	107±53	0.91	109±52	0.79
**TFAM**	174±94	*0.02*	192±91	*0.003*
**TFB1M**	193±63	*0.0005*	121±58	*<0.0005*
**TFB2M**	167±61	*0.004*	182±64	*0.0008*
**LON**	149±43	*0.005*	162±38	*<0.0005*
**CLPP**	150±52	*0.01*	153±49	*0.004*
**SPG7**	107±26	0.68	112±19	*0.18*
**YME1L1**	186±94	*0.02*	185±97	*0.004*

Data is given as mean and standard deviation. p for Student's t-test. ICU intensive care unit patient, ww wet weight, CS citrate synthase, SOD superoxide dismutase

However the groups in these post-hoc analyses are rather small and studies designed to study the temporal changes are needed. We tried to include a patient population with severe sepsis/septic shock induced multiple organ failure. Patients not recovering from an initial sepsis develop multiple organ failure requiring ICU treatment. The patients we included were not in their septic shock anymore and had developed multiple organ failure making them rather comparable. However a few patients with SOFA scores below 3 were included not fulfilling the criteria for multiple organ failure. Again post-hoc analyses comparing the ICU patients with SOFA score over 3 with the controls gave exactly the same changes and conclusions as before (Table 3 and 4). These post-hoc analyses show that this apparently heterogeneous group of ICU patients behave rather consistent with regard to mitochondrial changes and gene expression in skeletal muscle. Another possible confounding factor is the different treatments that the patients receive. When performing studies in actual ICU patients we are dealing with reality and this can not be changed for clinical reasons. Treatment with corticosteroids is the one most discussed in relation to muscle dysfunction. However when we compared the micro-array changes in our patients with the changes induced with methylprednisole treatment in rats only a 1–2% of the ICU regulated genes were affected in both the patients and the rat model [69], indicating that corticosteroid treatment in the ICU patients contributes little to the observed changes. However affects of other treatments can not be excluded.

Another limitation of the study is the isolation of mitochondria done for measuring the enzyme activities and the synthesis rates of mitochondrial protein. The sequential centrifugations used to isolate the mitochondria mainly isolate intact mitochondria and not the damaged mitochondria. Also this procedure isolated mainly the subsarcolemmal mitochondria from skeletal muscle and not the intramyofibrillar mitochondria. So when measuring the enzyme activities in the isolated mitochondria this represents mainly intact subsarcolemmal mitochondria. This selection of one subpopulation of the mitochondria is most likely also the explanation for the different values obtained for complex I and IV activities expressed per citrate synthase activity in the whole muscle and the isolated mitochondria. Also the increased complex IV activity in the isolated mitochondria and not in the whole muscle could be explained by this. Both the fractional synthesis rates of the mitochondria and the mitochondrial SOD activity can not be measured differently than in the isolated mitochondria. It is possible that the synthesis rates in the non-isolated mitochondria are decreased in the patients. However the gene expression data (both from the qPCR analyses and the micro-array) do support a normal synthesis as measured in the isolated mitochondria.

In summary, we demonstrated that while the skeletal muscle of septic ICU patients with multiple organ failure have reduced mitochondrial content, this is not associated with a gross failure in biogenesis of mitochondria since both *in vivo* protein synthesis and mitochondrial related mRNA abundance were sustained. However, loss of coordination of key elements of mitochondrial biogenesis was apparent, such that maturation of mitochondrial and mitochondrial networks may have still been compromised, resulting in activation of some specific mitochondrial matrix proteases. Further, in this first ever analysis of the global transcriptional responses in ICU patients, we find a substantial loss of muscle specific genes, a global oxidative stress response related to most probably cytokine signalling, altered insulin related signalling and a substantial overlap between patients and muscle wasting/inflammatory animal models. Failure to process mir-21 may have contributed to the activation of these signalling pathways, while such impairment hints at a wider problem with miRNA formation and hence control of tissue phenotype. Finally, we were able to demonstrate that the phenotype of skeletal muscle in ICU patients is not merely one of inactivity, it appears to be an actively remodelling tissue, influenced by several mediators, all of which may be open to manipulation with the aim to improve clinical outcome

## Supporting Information

Figure S1 Expression of mitochondrial transcription factors in C2C12 cells with and without insulin. Differentiated C2C12 cells were incubated with insulin from day 5 to day 6 (24 h) and expression of NRF2α, TFB1m and TFB2m were all measured by qPCR (white bars, water; grey bars, 0.6 µM insulin; black bars, 6 µM insulin). None of the mRNA levels were changed by insulin in relation to the controls. Values are given as mean±SEM.
(0.28 MB TIF)

Figure S2 NRF2α/GABP network. Ingenuity analyses of micro-array data indicated a network around NRF2α/GABP as significantly changed in muscle of the septic patients in comparison with age-matched controls. Gene descriptions can be found in supplemental data sheet S4. Red (and pink) means significantly increased gene expression and blue decreased. The ‘strength’ of the colour is an indication of how up regulated the gene was (with darker red being more up-regulated). Grey is a highly connected gene (to the transcript network that is altered) but itself is not altered at the mRNA level. Blue/Red hybrids are genes identified in the data base where the protein is actually at least two different genes and 1 sub-unit is down, while the other is up.
(0.72 MB TIF)

Figure S3 Comparisons of network analyses of atrogens in animal models and septic patients. Ingenuity network analyses including the atrogens defined by Golberg et al [32], [22] from catabolic and disuse animal models. The network build around these genes and related genes are shown. The changes in gene expression for the animal models are shown on the left and for the human data from the septic patients on the right. In large changes are similar except for the energy metabolism related genes which are downregulated in the animal models and not changed in the septic patients. An interesting observation is that this network is closely regulated by estradiol. Gene descriptions can be found in supplemental data sheet S3. Red (and pink) means significantly increased gene expression and blue decreased. The ‘strength’ of the colour is an indication of how up regulated the gene was (with darker red being more up-regulated). Grey is a highly connected gene (to the transcript network that is altered) but itself is not altered at the mRNA level. Blue/Red hybrids are genes identified in the data base where the protein is actually at least two different genes and 1 sub-unit is down, while the other is up.
(0.87 MB TIF)

Figure S4 Myc network. Ingenuity analyses of micro-array data indicated a network around MYC as most significantly changed in muscle of the septic patients in comparison with age-matched controls. Gene descriptions can be found in supplemental data sheet S4. Red (and pink) means significantly increased gene expression and blue decreased. The ‘strength’ of the colour is an indication of how up regulated the gene was (with darker red being more up-regulated). Grey is a highly connected gene (to the transcript network that is altered) but itself is not altered at the mRNA level. Blue/Red hybrids are genes identified in the data base where the protein is actually at least two different genes and 1 sub-unit is down, while the other is up.
(0.56 MB TIF)

Figure S5 Tissue remodelling networks. Ingenuity analyses of micro-array data indicated two network involved in tissue remodelling as highly significantly changed in muscle of the septic patients in comparison with age-matched controls. One network is build around TGFβ (a) and the other one around ESR1 (b). Gene descriptions can be found in supplemental data sheet S4. Red (and pink) means significantly increased gene expression and blue decreased. The ‘strength’ of the colour is an indication of how up regulated the gene was (with darker red being more up-regulated). Grey is a highly connected gene (to the transcript network that is altered) but itself is not altered at the mRNA level. Blue/Red hybrids are genes identified in the data base where the protein is actually at least two different genes and 1 sub-unit is down, while the other is up.
(0.83 MB TIF)

Supplemental data sheet S1 SAM results
(3.61 MB XLS)

Supplemental data sheet S2 Expression of mitochondrial genes from the micro-aray analysis.
(0.09 MB XLS)

Supplemental data sheet S3 Comparison of expression of atrogens identified in muscle of catabolic and disuse animal models (common to all models) and septic patients.
(0.05 MB XLS)

Supplemental data sheet S4 Gene network lists.
(0.10 MB XLS)

Supplemental data sheet S5 Canonical Network lists.
(0.06 MB XLS)
