# Correction: Dysregulation of Mitochondrial Dynamics and the Muscle Transcriptome in ICU Patients Suffering from Sepsis Induced Multiple Organ Failure

**DOI:** 10.1371/annotation/68d951f9-a236-472f-98af-24e4cc4c1a20

**Published:** 2008-12-12

**Authors:** Katarina Fredriksson, Inga Tjäder, Pernille Keller, Natasa Petrovic, Bo Ahlman, Camilla Schéele, Jan Wernerman, James A. Timmons, Olav Rooyackers

There was a sentence omitted from the Funding section. The complete funding information is as follows: These studies were supported by grants from the Swedish Research Council (grants 04210 and 14244), Karolinska Research Foundation, Karolinska University Hospital Research Funds and Heriot-Watt University Funds. The studies were also supported by an Affymetrix Translational Medicine Award (to JAT). None of the sponsors or funders were involved in design or conduct of the study or the manuscript.

